# Case Report: Multimodal treatment for two unresectable intraductal papillary mucinous neoplasms of the bile duct and literature review

**DOI:** 10.3389/fmed.2026.1726691

**Published:** 2026-05-07

**Authors:** Yinghao Fu, Yuhao Liang, Yuan Song, Lifan Chen, Xian Luo, Yewu Chen, Yi Xiao, Huadi Chen, Hao Xu

**Affiliations:** Department of Hepatobiliary Surgery, Affiliated Hospital of Guangdong Medical University, Zhanjiang, Guangdong, China

**Keywords:** case report, hepatic arterial infusion chemotherapy, intraductal papillary mucinous neoplasm of the bile duct, literature review, multimodal therapy, surufatinib, tislelizumab

## Abstract

Intraductal papillary mucinous neoplasm of the bile duct (IPMN-B) is a rare biliary tumor with marked heterogeneity. We report two cases of unresectable IPMN-B, both treated with gemcitabine plus oxaliplatin (Gemox)-based hepatic arterial infusion chemotherapy (HAIC) combined with tislelizumab and surufatinib. According to Response Evaluation Criteria in Solid Tumors version 1.1 (RECIST 1.1), calculated using the longest diameter of the target lesion, case 1 achieved radiographic stable disease (SD) and improvement in laboratory indices after two cycles of HAIC combined with targeted immunotherapy, followed by one cycle of maintenance therapy with tislelizumab plus surufatinib. Case 2 achieved radiographic partial response (PR) after four cycles of treatment, accompanied by marked improvement in abdominal pain and cholestatic indices. No severe treatment-related adverse events occurred in either patient, and both remained alive at the last follow-up. To place these observations in the context of existing evidence, we further reviewed the epidemiology, clinical manifestations, pathological and imaging features, treatment strategies, and prognosis of IPMN-B based on the available literature. These two cases suggest that multimodal combination therapy may have potential value in unresectable IPMN-B, although the durability of response, long-term safety, and the subgroups most likely to benefit still require further study.

## Introduction

Intraductal papillary mucinous neoplasm of the bile duct (IPMN-B), also commonly referred to as intraductal papillary neoplasm of the bile duct (IPNB), is a rare biliary epithelial neoplasm. According to the 5th edition of the World Health Organization (WHO) Classification of Digestive System Tumours (2019), it is defined as a grossly visible premalignant neoplasm characterized by intraductal papillary or villous growth of biliary-type epithelium, with lesions ranging from low-grade to high-grade dysplasia and, in some cases, an associated invasive carcinoma (1, 2). Although it shares morphological and molecular similarities with intraductal papillary mucinous neoplasm of the pancreas and is often regarded as its biliary counterpart, its clinicobiological behavior remains distinct from that of conventional cholangiocarcinoma (3).

Compared with conventional cholangiocarcinoma, IPMN-B is often associated with a more favorable prognosis, but it remains clinically heterogeneous and has definite malignant potential (4, 5). Improvements in magnetic resonance imaging/magnetic resonance cholangiopancreatography (MRI/MRCP), endoscopic retrograde cholangiopancreatography (ERCP), and cholangioscopy have enhanced recognition of this disease; however, because IPMN-B is uncommon, evidence regarding optimal management—particularly for unresectable disease—remains limited.

Surgical resection with negative margins remains the standard curative treatment. For patients who are not candidates for surgery, however, there is still no widely accepted standard regimen that is both effective and well tolerated.

Here, we report two patients with unresectable IPMN-B who were treated with gemcitabine plus oxaliplatin (Gemox)-based hepatic arterial infusion chemotherapy (HAIC) in combination with tislelizumab and surufatinib after multidisciplinary evaluation. Both patients experienced radiographic and biochemical improvement. To place these observations in clinical context, we provide a focused review of the literature on key diagnostic features, current treatment options, and the limitations of this exploratory approach.

## Case description

### Case 1

#### Patient information and clinical presentation

**Figure 1:**
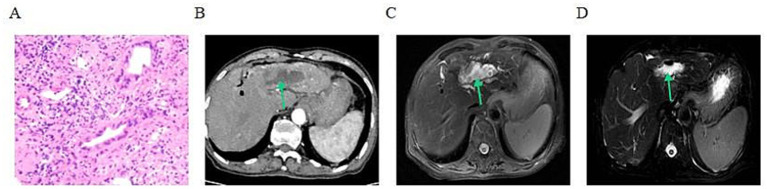
Pathologic, computed tomography, and magnetic resonance imaging findings of case 1. **(A)** Ultrasound-guided liver biopsy specimen stained with hematoxylin and eosin (H&E), showing papillary growth of tumor cells with mild nuclear atypia (original magnification, ×200). **(B)** Baseline axial contrast-enhanced computed tomography image in the arterial phase, showing the biliary lesion (green arrow). **(C)** Baseline axial respiratory-triggered fat-suppressed T2-weighted PROPELLER magnetic resonance image obtained on April 25, 2025, showing the biliary lesion (green arrow). **(D)** Follow-up axial T2-weighted SPIR liver magnetic resonance image obtained on July 11, 2025, after 2 cycles of Gemox-HAIC and 2 cycles of tislelizumab plus surufatinib, showing interval reduction of the lesion after treatment (green arrow). PROPELLER, Periodically Rotated Overlapping Parallel Lines with Enhanced Reconstruction; SPIR, Spectral Presaturation with Inversion Recovery; MRI, magnetic resonance imaging; CT, computed tomography; IPMN-B, intraductal papillary mucinous neoplasm of the bile duct; Gemox, gemcitabine plus oxaliplatin; HAIC, hepatic arterial infusion chemotherapy.

A 79-year-old man was referred in May 2025 after a liver lesion had been detected on routine examination 10 days earlier. He had undergone surgery for gallstones about 50 years previously and had been treated for a urinary tract infection 3 months before admission. There was no relevant family history. He had no history of hypertension, diabetes, coronary artery disease, hepatitis, tuberculosis, smoking, or alcohol use. On admission, he had mild scleral icterus without abdominal pain, distension, nausea, or vomiting. Ultrasound-guided liver biopsy showed papillary growth with mild nuclear atypia. Immunohistochemistry supported a biliary epithelial neoplasm with intestinal differentiation and argued against hepatocellular origin (Figure 1A). Baseline CT showed a hilar/intrahepatic biliary lesion with biliary dilatation (Figure 1B). MRI showed an intrahepatic biliary lesion measuring approximately 42 × 49 × 27 mm, unclear visualization of the left portal vein branch, multiple stones in both hepatic lobes, and intra and extrahepatic bile duct dilatation (Figure 1C). Taken together, these findings supported IPMN-B with low-grade intraepithelial neoplasia, predominantly of intestinal type.

#### Diagnostic assessment

The diagnosis in this case was based on an integrated assessment of imaging, histopathology, and immunohistochemistry. Pre-admission MRI showed an intrahepatic mass with multiple stones in both hepatic lobes and intrahepatic and extrahepatic bile duct dilatation, raising initial concern for a biliary malignancy. However, imaging alone could not reliably distinguish conventional intrahepatic cholangiocarcinoma, an intraductal papillary neoplasm, and stone-related inflammatory biliary disease, and ultrasound-guided biopsy of the liver mass was therefore performed. Pathology revealed papillary growth of tumor cells with mild nuclear atypia. Immunohistochemistry showed positivity for cytokeratin 7 and cytokeratin 19, supporting biliary epithelial origin; positivity for caudal type homeobox 2 and cytokeratin 20, suggesting intestinal differentiation; focal positivity for mucin 5 AC and mucin 6; a Ki-67 labeling index of approximately 40% in hotspot areas; and negativity for Arginase-1, arguing against a hepatocellular lesion. In combination with the histomorphology, these findings favored IPMN-B with low-grade intraepithelial neoplasia, predominantly of the intestinal subtype. The main diagnostic challenge was the absence of typical symptoms together with coexisting biliary stones and ductal dilatation, which increased the difficulty of judging the nature and extent of the lesion. From a prognostic standpoint, the patient’s advanced age, poor visualization of the left portal vein branch, and cholestatic manifestations suggested locally complex disease requiring close follow-up and reassessment. After multidisciplinary evaluation, curative surgery was not pursued because of advanced age, locally complex biliary involvement, and limited feasibility of curative margin-negative resection.

#### Therapeutic intervention

The patient received two 3-week cycles of Gemox-based HAIC combined with tislelizumab and surufatinib. The regimen consisted of gemcitabine 1,000 mg/m^2^ and oxaliplatin 100 mg/m^2^. A 5-Fr arterial access was established via the femoral artery using the Seldinger technique, followed by superselective catheterization of the tumor-feeding artery. Digital subtraction angiography confirmed catheter placement within the dominant feeding branch. Gemcitabine was diluted in 100 mL normal saline and infused with a microinfusion pump within 1 h; oxaliplatin was diluted in 250 mL of 5% glucose solution and infused within 3 h. Tislelizumab 200 mg was administered intravenously every 3 weeks, and surufatinib was given orally at 100 mg once daily. After completion of two cycles, the patient received one additional cycle of maintenance therapy with tislelizumab plus surufatinib. No dose reduction, treatment delay, or interruption occurred because of toxicity.

#### Follow-up and outcomes

Serologic parameters improved after treatment: carbohydrate antigen 19–9 (CA19-9) decreased from 39.30 U/mL to 27.5 U/mL, and alkaline phosphatase (ALP) decreased from 160.0 U/L to 95.9 U/L. After two treatment cycles, repeat MRI showed a reduction in lesion size to 41 × 41 × 36 mm (Figure 1D), which was assessed as stable disease according to Response Evaluation Criteria in Solid Tumors version 1.1 (RECIST 1.1). Clinically, the patient remained in good general condition, without new abdominal pain, fever, or worsening obstruction. He completed the planned two cycles of HAIC-based combination therapy followed by one cycle of maintenance therapy, and no grade ≥3 treatment-related adverse events were observed. At the last follow-up on August 14, 2025, he was still alive but did not continue treatment for financial reasons.

### Case 2

#### Patient information and clinical presentation

**Figure 2:**
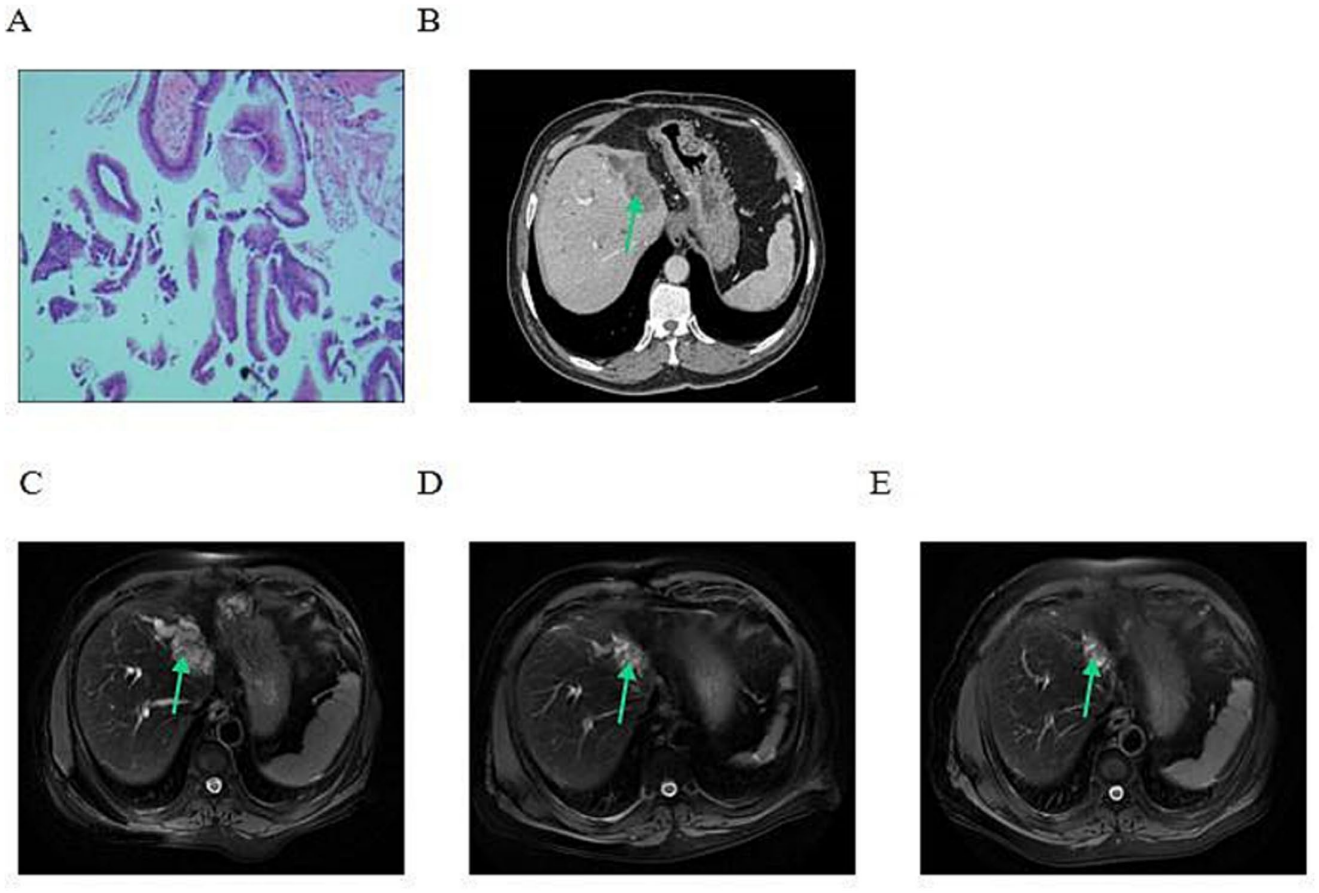
Pathologic, computed tomography, and magnetic resonance imaging findings of case 2. **(A)** Endoscopic biopsy specimen stained with hematoxylin and eosin (H&E), showing papillary epithelial proliferation consistent with intraductal papillary mucinous neoplasm of the bile duct (original magnification, ×100). **(B)** Baseline axial contrast-enhanced computed tomography image in the portal venous phase, showing the biliary lesion (green arrow). **(C)** Baseline axial respiratory-triggered fat-suppressed T2-weighted PROPELLER magnetic resonance image obtained on October 2, 2024, showing the biliary lesion (green arrow). **(D)** Axial respiratory-triggered fat-suppressed T2-weighted PROPELLER magnetic resonance image obtained on December 19, 2024, after 2 cycles of Gemox-HAIC combined with tislelizumab and surufatinib, showing interval tumor shrinkage (green arrow). **(E)** Axial respiratory-triggered fat-suppressed T2-weighted PROPELLER magnetic resonance image obtained on February 18, 2025, after 4 cycles of Gemox-HAIC combined with tislelizumab and surufatinib, showing persistent reduction of the lesion compared with baseline (green arrow). PROPELLER, Periodically Rotated Overlapping Parallel Lines with Enhanced Reconstruction; MRI, magnetic resonance imaging; CT, computed tomography; IPMN-B, intraductal papillary mucinous neoplasm of the bile duct; Gemox, gemcitabine plus oxaliplatin; HAIC, hepatic arterial infusion chemotherapy.

A 68-year-old man presented in September 2024 with intermittent upper abdominal pain for 10 days that had worsened over the preceding 3 days. Ten years earlier, he had undergone left lateral hepatectomy and common bile duct exploration for common bile duct stones and intrahepatic bile duct stones. There was no relevant family history. He had no history of hypertension, diabetes, coronary artery disease, hepatitis, tuberculosis, smoking, or alcohol use. On admission, he reported right upper abdominal pain and vomiting, with scleral icterus and dark urine. ERCP with endoscopic biopsy was performed. The specimen from fine-needle biopsy of the common bile duct was limited, but pathology was consistent with IPMN-B; immunohistochemistry could not be completed because of insufficient tissue (Figure 2A). Baseline CT showed a hilar/intrahepatic biliary lesion with associated biliary abnormalities (Figure 2B). MRI demonstrated an intrahepatic biliary lesion measuring about 50 × 33 mm, common bile duct stones with cholangitis, and intra and extrahepatic bile duct dilatation (Figure 2C).

#### Diagnostic assessment

The diagnosis in this case was based on a combined assessment of imaging, ERCP findings, and pathological biopsy. Admission MRI showed an intrahepatic mass with common bile duct stones, cholangitis, and intrahepatic and extrahepatic bile duct dilatation, initially suggesting biliary malignancy. However, imaging alone could not reliably distinguish conventional intrahepatic cholangiocarcinoma, an intraductal papillary neoplasm, and biliary inflammatory disease related to stones or infection. ERCP with endoscopic biopsy of the biliary lesion was therefore performed. Pathology was consistent with IPMN-B, but because the fine-needle biopsy specimen from the common bile duct was limited, further immunohistochemical evaluation could not be performed, which also restricted more detailed assessment of subtype and possible invasive components. The main diagnostic challenge was the coexistence of common bile duct stones, cholangitis, and bile duct dilatation, producing clinical and imaging findings that overlapped with obstructive inflammatory disease. On the basis of the patient’s symptoms, imaging findings, and pathology, a final diagnosis of IPMN-B was made. Prognostically, the presence of jaundice, cholangitis, and biliary obstruction indicated locally complex disease requiring continued dynamic assessment during subsequent treatment using both imaging and clinical findings. After multidisciplinary evaluation, curative surgery was not pursued because of locally complex biliary disease in the setting of prior hepatobiliary surgery, cholangitis, and biliary obstruction, with limited feasibility of achieving meaningful margin-negative resection.

#### Therapeutic intervention

The patient received a combination regimen similar to that used in case 1, for a total of four cycles. Chemotherapy and HAIC were administered in the same manner as in case 1. Tislelizumab 200 mg was given intravenously each cycle, and surufatinib 100 mg was administered orally once daily. Overall adherence during treatment was good, and the patient completed all four planned cycles. No dose adjustment, cycle delay, or early discontinuation was required because of toxicity.

#### Follow-up and outcomes

Laboratory indices improved markedly after treatment: total bilirubin decreased from 40.7 μmol/L to 8.2 μmol/L; CA19-9 decreased from 21.30 U/mL to 8.74 U/mL; carcinoembryonic antigen (CEA) decreased from 29.40 U/mL to 0.67 U/mL; and ALP decreased from 185.6 U/L to 91.6 U/L. After two cycles, repeat MRI showed tumor shrinkage to 14 × 16 mm (Figure 2D), and the response was assessed as partial response. After a total of four cycles, MRI showed further shrinkage to 13 × 7 mm (Figure 2E), indicating sustained partial response. The patient’s abdominal pain was clearly relieved, suggesting meaningful subjective symptom benefit. No significant treatment-related adverse events were observed during the entire treatment course, particularly no grade ≥3 treatment-related adverse events or unexpected serious complications. At the last follow-up on March 19, 2025, the patient remained alive but likewise declined further treatment for financial reasons.

**Figure 3:**
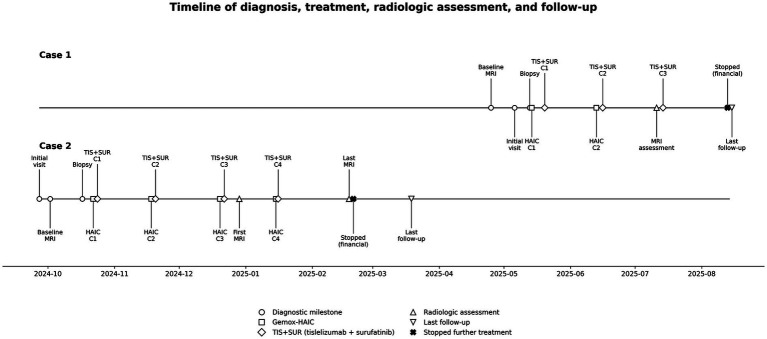
Schematic timeline from diagnosis through treatment and follow-up in two patients with unresectable IPMN-B. Key clinical milestones for cases 1 and 2 are shown, including initial presentation, pathological confirmation, Gemox-HAIC treatment, administration of tislelizumab plus surufatinib, radiographic response assessment, and last follow-up. Case 1 did not continue further treatment for financial reasons on August 14, 2025; case 2 declined further treatment for financial reasons on March 19, 2025. IPMN-B, intraductal papillary mucinous neoplasm of the bile duct; Gemox, gemcitabine plus oxaliplatin; HAIC, hepatic arterial infusion chemotherapy.

The main diagnostic course, treatment process, imaging response assessments, and follow-up time points for both patients are summarized in Figure 3.

## Discussion

This study reports two cases of unresectable intraductal papillary mucinous neoplasm of the bile duct (IPMN-B). After multidisciplinary evaluation, both patients received Gemox-based HAIC combined with tislelizumab and surufatinib and achieved varying degrees of radiographic response together with improvement in laboratory parameters. Unlike previous studies that have focused mainly on resectable disease, the present report is centered on whether a multimodal treatment pathway may merit further exploration in patients who have lost the opportunity for curative resection.

Evidence specifically addressing systemic treatment for unresectable IPMN-B is extremely limited. From the broader perspective of biliary tumor management, however, local treatment alone or palliative drainage is often insufficient to achieve local tumor control, relief of biliary symptoms, and potential control of systemic disease at the same time. In both patients, multidisciplinary evaluation determined that curative resection was not feasible, and the local disease was anatomically and clinically complex; accordingly, the therapeutic focus shifted toward local tumor control, relief of biliary symptoms, and delay of disease progression.

In these two patients, the main clinical problems were concentrated in the local biliary tumor burden, bile duct dilatation, and cholestatic manifestations; case 2 also had cholangitis and pronounced abdominal pain. Accordingly, treatment had to pursue not only systemic disease control but also relatively rapid improvement in local biliary disease and related symptoms. Gemox, as one of the commonly used chemotherapy backbones in biliary tract malignancies, was therefore a practical locoregional partner. When combined with HAIC, it can increase local drug exposure through the hepatic arterial route and may theoretically strengthen control of biliary lesions and improve local cytoreductive efficiency (6). At the same time, local treatment alone or palliative drainage is often insufficient to address potential occult systemic disease. We therefore added the programmed cell death protein 1 (PD-1) inhibitor tislelizumab and surufatinib, which has both anti-angiogenic and immunomodulatory properties, with the aim of integrating local cytoreduction, systemic disease control, and modulation of the tumor microenvironment. In theory, surufatinib and tislelizumab may exert complementary effects at different levels by attenuating the immunosuppressive barrier on the one hand and enhancing T-cell-mediated cytotoxicity on the other (7–9).

Available studies suggest that immune microenvironment alterations may already occur at an early stage of IPMN-B-associated biliary carcinogenesis, particularly with reduced intraepithelial cluster of differentiation 8-positive (CD8^+^) T-cell infiltration (10). In addition, PD-1 axis-related immune escape has also been reported in cholangiocarcinoma (11). More broadly, studies in biliary tract tumors have shown that immune checkpoint inhibitor-based chemoimmunotherapy can provide survival benefit, while combinations of immunotherapy, antiangiogenic therapy, and Gemox have also demonstrated clinical activity (12, 13). Therefore, the incorporation of immunotherapy into the multimodal strategy used in this study should be regarded primarily as an exploratory attempt supported by the broader biliary tract cancer literature and the pathobiological features of IPMN-B, rather than as an established approach backed by high-level IPMN-B-specific evidence.

It should be emphasized that we did not obtain paired tumor tissue samples before and after treatment, nor did we assess CD8^+^ T-cell infiltration, interferon-*γ*, or other indicators of the immune microenvironment. Therefore, we are unable to determine whether the observed clinical benefit was mediated through enhanced antitumor immunity. We only observed improvements in imaging findings, laboratory parameters, and symptoms at the clinical level, which suggests that this combination regimen may confer benefit.

### Review of the literature

IPMN-B is now regarded as a distinct biliary neoplasm with substantial clinicopathologic heterogeneity (1, 2, 14–17). Available series suggest that it accounts for approximately 9%–10% of resectable biliary tract tumors and is reported more often in East Asia, usually in older men (14, 15, 18). More recently, the EUR-IPMN-B study provided multicenter data from European centers, expanding the available evidence beyond the predominantly East Asian literature (19). A subsequent comparative analysis between European and Nagoya cohorts also suggested potential geographic differences in patient background, tumor characteristics, and management patterns (20). Clinical presentation is variable and may include abdominal pain, jaundice, cholangitis, or incidental detection on imaging (5, 15, 18, 21–23). Laboratory abnormalities such as elevated ALP, bilirubin, CA19-9, or CEA may be present, particularly when obstructive or invasive components coexist, but these findings are nonspecific (24, 25). Our two patients reflected this heterogeneity well: case 1 was detected incidentally and remained relatively oligosymptomatic, whereas case 2 presented with abdominal pain, jaundice, cholangitis, and biliary obstruction.

From a pathological perspective, IPMN-B is characterized by papillary epithelial proliferation with mucin secretion and variable degrees of dysplasia, but it is not a single biologic entity (2, 16, 17). Current classifications distinguish type 1 and type 2 lesions, which differ in architecture, immunophenotype, molecular alterations, and prognosis (16, 17, 26, 27). This heterogeneity is clinically relevant because some lesions follow a relatively indolent intraductal course, whereas others already harbor invasive components at diagnosis or progress toward a phenotype resembling conventional cholangiocarcinoma (17, 26, 27). Reported rates of malignant transformation or associated invasion are substantial, which helps explain why surgical management is generally recommended whenever resection remains feasible (14, 27, 28).

Imaging assessment is therefore central to diagnosis and treatment planning. MRI/MRCP can help distinguish mural nodules from intraductal mucin, diffusion-weighted sequences may increase lesion conspicuity, and characteristic findings such as the thread sign or hepatobiliary-phase hypointensity on gadoxetic acid-enhanced MRI may support the diagnosis (29–33). Nevertheless, no single modality is definitive. ERCP remains useful for biliary decompression and tissue acquisition, but visualization may be limited by mucus, and pathological sampling may be insufficient for full immunophenotypic or molecular characterization, as occurred in case 2 (34–36).

These diagnostic considerations are directly relevant to the present report. In case 1, the lesion was discovered incidentally in the setting of hepatolithiasis and biliary dilatation, and tissue diagnosis together with immunohistochemistry was important for distinguishing IPMN-B with intestinal differentiation from other biliary or hepatocellular lesions. In case 2, the coexistence of choledocholithiasis, cholangitis, and obstructive jaundice created a more overt clinical picture, but the biopsy sample remained limited and did not permit full immunophenotypic assessment. Together, the two cases illustrate the broad clinical spectrum of IPMN-B and the practical reality that diagnosis often depends on integrating imaging, endoscopic findings, pathology, and the overall clinical course rather than relying on any single test. The major findings from representative studies on the clinicopathological features, diagnosis, and treatment of IPMN-B are summarized in Table 1.

**Table 1:** Major findings from representative studies on the clinicopathological features, diagnosis, and treatment of IPMN-B.

Topic	Representative references	Main findings	Relevance to the present study
Disease definition and classification	Chen et al (1); Bosman et al (2); Rocha et al (14).	IPMN-B has gradually been established as an independent pathological entity and is regarded as one of the biliary counterparts of pancreatic IPMN.	Provides the basis for disease naming and conceptual definition in the present report.
Clinical features and epidemiology	Wang et al (5); Kubota et al (15); Jung et al (22).	IPMN-B is rare, occurs more commonly in East Asian populations, and often presents with abdominal pain, jaundice, or cholangitis, although it may also be asymptomatic.	Consistent with the incidental detection of case 1 and the presentation of case 2 with abdominal pain and cholangitis.
Pathological subtypes and biological behavior	Nakanuma et al (16, 17); Sclabas et al (26); Zen et al (27).	IPMN-B can be divided into type 1 and type 2 lesions, with differences in histology, immunophenotype, molecular features, and invasiveness among subtypes.	Supports the discussion of intestinal differentiation, pathological heterogeneity, and malignant potential in the present study.
Imaging diagnosis	Park et al (3); Li et al (29); Kraus et al (30); Hong et al (31).	MRI/MRCP is superior to CT for depicting mucin, mural nodules, and bile duct dilatation, and some MRI signs may be diagnostically informative.	Supports the use of MRI as the principal imaging modality in the present report.
Value of ERCP/cholangioscopy	Tsuyuguchi et al (34); Perez-Cuadrado-Robles et al (36).	ERCP can be used for biliary sampling, although mucin may compromise interpretation; cholangioscopy offers direct visualization and facilitates targeted biopsy.	Echoes the diagnostic pathway in case 2, in which the diagnosis was established by ERCP-guided biopsy.
Curative treatment and prognosis	Matsumoto et al (23); D’Souza et al (28).	Surgical resection remains the main curative treatment, and R0 resection is the key prognostic determinant.	Supports the core judgment in this report that surgery remains the principal curative treatment for IPMN-B.
Nonsurgical treatment for unresectable disease	Valente et al (41); Chi et al (42); Arai et al (43); Vogel et al (44).	Published case-based evidence suggests that PDT and APC may be used for local control or palliation. For unresectable disease, systemic treatment in clinical practice is largely extrapolated from the broader biliary tract cancer framework, but IPMN-B-specific evidence remains very limited.	Provides the background rationale for exploring Gemox-HAIC combined with tislelizumab and surufatinib in the present study.
Potential contribution of the present cases	This study	Both patients with unresectable IPMN-B achieved radiographic and laboratory improvement after multimodal combination therapy, without severe treatment-related adverse events.	Suggests that multimodal combination therapy may warrant exploration in unresectable IPMN-B, although prospective validation is still needed.

IPMN-B, intraductal papillary mucinous neoplasm of the bile duct; IPMN, intraductal papillary mucinous neoplasm; MRI/MRCP, magnetic resonance imaging/magnetic resonance cholangiopancreatography; CT, computed tomography; ERCP, endoscopic retrograde cholangiopancreatography; R0, microscopically margin-negative; PDT, photodynamic therapy; APC, argon plasma coagulation; Gemox, gemcitabine plus oxaliplatin; HAIC, hepatic arterial infusion chemotherapy.

Surgical resection remains the cornerstone of curative treatment, and microscopically margin-negative (R0) resection is the most important prognostic goal (5, 22, 23, 28, 37–39). Recent case-based literature reviews have likewise continued to emphasize that, whenever patients are suitable surgical candidates, curative resection should be pursued whenever possible (40). The specific surgical procedure generally depends on tumor location: intrahepatic lesions often require hepatectomy, whereas hilar or distal lesions may require bile duct resection combined with pancreatoduodenectomy (5, 23, 28, 37–39). Even lesions considered noninvasive are often managed actively because of their malignant potential, the possibility of occult invasion, and the tendency toward multifocal or progressive disease (27, 28). For unresectable disease, however, the available evidence remains limited. Reported nonoperative approaches have mainly included biliary drainage, chemoradiotherapy, photodynamic therapy, and argon plasma coagulation for local control or palliation; although these modalities may provide some benefit in selected settings, they are still insufficient to define a standard systemic strategy for unresectable IPMN-B (41–44). Against this background, the responses observed in our two patients are insufficient to establish efficacy, but they suggest that a multimodal strategy integrating locoregional treatment, immunotherapy, and antiangiogenic therapy may warrant further investigation in selected unresectable cases characterized by dominant local biliary tumor burden, cholestatic symptoms, and the absence of a clear curative surgical option.

## Limitations

Several limitations should be acknowledged. First, this report includes only two patients from a single center, which limits the strength and generalizability of the findings. Second, follow-up was relatively short and insufficient to assess long-term progression-free survival, overall survival, or recurrence patterns. Third, because systematic molecular classification, cancer genomic profiling, dynamic biological assessments, and evaluation of the tumor microenvironment were unavailable, the relative contribution of each treatment component and the subgroups most likely to benefit remain uncertain. In addition, the lack of a control group precludes direct comparison with chemotherapy alone, biliary drainage, or other local treatment strategies. These findings should therefore be interpreted as exploratory rather than as evidence establishing a treatment standard.

### Conclusion and future directions

IPMN-B is a rare and heterogeneous biliary tumor that is now recognized as an independent clinicopathological entity. Surgical resection remains the only widely accepted curative treatment, and R0 resection is the key determinant of prognosis. Although advances in imaging, cholangioscopy, and pathological classification have improved diagnostic precision and risk stratification, evidence to guide treatment of unresectable IPMN-B remains limited.

In this report, both patients showed tumor regression or durable disease control, with concurrent improvement in symptoms or laboratory indices, after Gemox-based HAIC combined with tislelizumab and surufatinib. These findings suggest that a multimodal strategy integrating interventional therapy, immunotherapy, and antiangiogenic therapy may be worth further study in selected unresectable cases. However, larger prospective studies with longer follow-up are still needed to define its durability, safety, and the patients most likely to benefit.

## Data Availability

The raw data supporting the conclusions of this article will be made available by the authors, without undue reservation.
